# Pnc1 piggy-back import into peroxisomes relies on Gpd1 homodimerisation

**DOI:** 10.1038/srep42579

**Published:** 2017-02-17

**Authors:** Nadal A. Al Saryi, John D. Hutchinson, Murtakab Y. Al-hejjaj, Svetlana Sedelnikova, Patrick Baker, Ewald H. Hettema

**Affiliations:** 1 Department of Molecular Biology and Biotechnology University of Sheffield Firth Court, Western Bank Sheffield S10 2TN United Kingdom

## Abstract

Peroxisomes are eukaryotic organelles that posttranslationally import proteins via one of two conserved peroxisomal targeting signal (PTS1 or 2) mediated pathways. Oligomeric proteins can be imported via these pathways but evidence is accumulating that at least some PTS1-containing monomers enter peroxisomes before they assemble into oligomers. Some proteins lacking a PTS are imported by piggy-backing onto PTS-containing proteins. One of these proteins is the nicotinamidase Pnc1, that is co-imported with the PTS2-containing enzyme Glycerol-3-phosphate dehydrogenase 1, Gpd1. Here we show that Pnc1 co-import requires Gpd1 to form homodimers. A mutation that interferes with Gpd1 homodimerisation does not prevent Gpd1 import but prevents Pnc1 co-import. A suppressor mutation that restores Gpd1 homodimerisation also restores Pnc1 co-import. In line with this, Pnc1 interacts with Gpd1 *in vivo* only when Gpd1 can form dimers. Redirection of Gpd1 from the PTS2 import pathway to the PTS1 import pathway supports Gpd1 monomer import but not Gpd1 homodimer import and Pnc1 co-import. Our results support a model whereby Gpd1 may be imported as a monomer or a dimer but only the Gpd1 dimer facilitates co-transport of Pnc1 into peroxisomes.

Peroxisomes are organelles that participate in a large number of cellular processes including fatty acid oxidation and other hydrogen peroxide-producing oxidation reactions. Enzymes destined for peroxisomes are synthesized in the cytosol and are posttranslationally imported^1^. Most enzymes contain a Peroxisome Targeting Signal type 1 (PTS1) that comprises a c-terminal tripeptide with the sequence serine-lysine-leucine (SKL) or a derivative thereof^2^ which is recognised by Pex5^3^. A minority of imported proteins lack a PTS1, but do contain a different type of targeting signal, namely a PTS2. A PTS2 comprises a nonapeptide found near the amino terminus of these peroxisomal proteins^4^. The PTS2 is recognized by Pex7 and a coreceptor (Pex18 or Pex21 in *Saccharomyces cerevisiae,* Pex20 in other fungi) in the cytosol^5^,^6^,^7^,^8^,^9^,^10^,^11^,^12^,^13^.

Cargo-receptor complexes dock onto the peroxisomal membrane docking complex Pex13/Pex14/Pex17^14^,^15^, which initiates formation of a transient pore. Two distinct pores have been described that mediate either PTS1 or PTS2 protein translocation. A unique feature of these pores is that the cargo-loaded PTS1 receptor or PTS2 coreceptors integrate into the membrane and become integral parts of it^16^,^17^. The import pores are considered to be highly dynamic structures that transiently assemble to accommodate the translocation of a wide range of cargoes including folded and oligomeric proteins^18^. After cargo translocation the receptors and coreceptors are ubiquitinated and recycled to the cytosol via an ATP-dependent extraction process that depends on the AAA+ proteins Pex1 and Pex6^19^,^20^,^21^. Various recent articles review different aspects of our current understanding of how proteins are targetted and translocated across the membrane^22^,^23^,^24^,^25^,^26^,^27^.

Although import of oligomers has been widely reported (for review see refs 11, 28 and 29), oligomers may not be the preferred substrate for the PTS1 import route as for several abundant peroxisomal proteins it was shown that Pex5 binding prevents oligomerisation *in vitro*^30^,^31^. This suggests that Pex5 is both acting as a targeting receptor and a molecular chaperone that regulates oligomerisation^29^.

Not all peroxisomal matrix proteins contain a PTS1 or PTS2. Some of these proteins are imported by piggy-backing on a different, PTS1-containing, matrix protein. The two examples of these, superoxide dismutase and lactate dehydrogenase both display partially peroxisomal localisation and cytosolic localisation^32^,^33^. Another example of hetero-oligomeric import is Pyrazinamidase/Nictonamidase I (Pnc1), which is targetted via the PTS2 pathway to peroxisomes but itself lacks a PTS2^34^. Pnc1 is itself an oligomeric protein. Pnc1 activity was found associated with homomultimers and *in vitro* it has a propensity to self-associate into helical arrays^35^,^36^. Pnc1 functions in the NAD^+^ salvage pathway by converting nicotinamide to nicotinic acid. During the course of our study, two independent groups reported that Pnc1 is co-imported with the PTS2-containing protein Gpd1 (glycerol 3-phosphate dehydrogenase 1)^37^,^38^.

Gpd1 is required for the production of glycerol from dihydroxyacetone phosphate during hyperosmotic stress. Glycerol acts as an osmolyte and allows cells to grow under high salt conditions^39^.

Here we present our analysis of Gpd1 import and the requirements of peroxisomal co-import of Pnc1. We find that Gpd1 can be imported as a dimer via the PTS2 pathway and that a mutant that is affected in dimer formation is still imported. Switching Gpd1 from the PTS2 pathway to the PTS1 pathway restores its import in mutants disturbed in PTS2 protein import but only monomeric import is then observed. Piggy-back import of Pnc1 is neither supported by Gpd1 following the PTS1 pathway nor by the Gpd1 dimerisation mutant. However, a double mutation that restores Gpd1 homodimer formation also restores Pnc1 piggy-back import. These data imply that Pnc1 piggy-back import relies on dimerisation of Gpd1.

## Results

### Gpd1 can be imported as monomer and dimer

Previous studies have shown that the PTS2-containing enzyme Gpd1 is partially imported into peroxisomes and that this localization requires the cytosolic PTS2 receptor Pex7 and its coreceptor Pex21^38^,^40^. Indeed, Gpd1-GFP partially colocalises with the red fluorescent peroxisomal marker protein HcRed-PTS1 in WT cells but not in *pex7* and *pex21* cells (Fig. S1A). In *pex5* cells, Gpd1-GFP is located to puncta that do not contain HcRed-PTS1 as in this mutant PTS1 import is selectively blocked. Since deletion of the PTS2 (ΔN-Gpd1) prevented import in *gpd1* cells but not in WT cells, it was suggested that Gpd1 dimerises before import and that ΔN-Gpd1 piggy-backs on endogenous Gpd1 in WT cells^40^. In line with this, the crystal structure of both human Gpd1 and *Saccharomyces cerevisiae* Gpd1 revealed a dimeric organization^41^,^42^.

**Figure 1:**
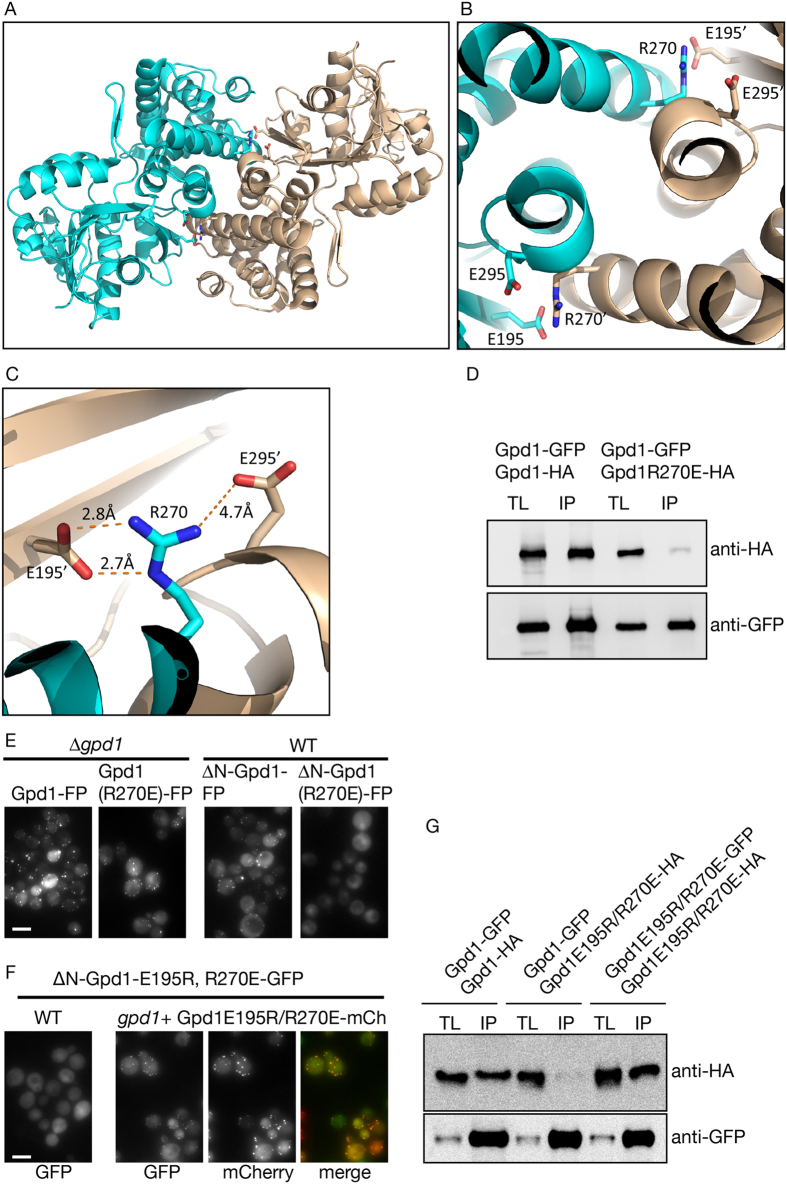
Gpd1 can be imported as a monomer and homodimer. (**A–C**) A cartoon representation of the dimer of *S. cerevisiae* glycerol-3-phosphate dehydrogenase (4FGW). (**A**) A cartoon representation, viewed down the two fold axis, with each subunit highlighted (blue, brown). (**B**) A close up of the same view, highlighting the ion-pair interaction formed as R270 from one subunit packs between E195 and E295 from the other, side chains drawn as sticks (oxygen, red; nitrogen, blue). (**C**) Further close up showing how R270 from one subunit (blue) packs between E195 and E295 from the other (light brown). Hydrogen bonds shown in orange with distances in Å. (**D**) Co-immunoprecipitation experiments using cell lysates from *gpd1* cells expressing Gpd1-GFP and either Gpd1-HA or Gpd1R270E-HA. Cells were grown on 1M NaCl containing medium. Proteins were detected by immunoblotting. TL, total lysate; IP, immunoprecipitate. (**E**) Localisation of Gpd1-GFP, Gpd1R270E-GFP in *gpd1* cells and of ΔN-Gpd1-GFP and ΔN-Gpd1R270E-GFP in WT cells. (**F**) Localisation of ΔN-Gpd1E195R, R270E-GFP in WT cells (panel 1) and *gpd1* cells also expressing Gpd1E195R, R270E-mCherry (panel 2–4). (**G**) Co-immunoprecipitation experiments using cell lysates from *gpd1* cells co-expressing either Gpd1-GFP or Gpd1E195R, R270E-GFP and either Gpd1-HA or Gpd1 E195R, R270E-HA. (**E,F**) Fluorescence microscopy of cells from cultures in log phase. All versions of Gpd1 were expressed from centromeric plasmids under control of endogenous promoter. Bar, 5 μm.

Analysis of the Gpd1 structure from *S. cerevisiae* (4FGW)^41^, using the program AreaImol (CCP4)^43^, showed that the protein forms a dimer, with 11% of the subunit accessible surface buried in the dimer interface (Fig. 1A). This interface is constructed from a mix of hydrophobic and hydrophilic interactions and contains two ion pair interactions, each consisting of the side chain of R270 from one subunit packing between E195 (2.8 Å) and E295 (4.7 Å) from the other subunit (Fig. 1B,C). These residues are conserved in human Gpd1, which also forms a dimer, as E163, R229 and E254 (Fig. S1B,C)^42^. As these residues form the only charged interaction in the dimer interface, we speculated that replacement of the central arginine with a glutamate (R270E), would severely disrupt the interface, and perhaps prevent dimerization. We tagged Gpd1 with either GFP or HA at the carboxy-terminus and co-expressed these in *gpd1* cells. Co-immunoprecipitation experiments confirmed that Gpd1 but not Gpd1R270E can form homo-oligomers *in vivo* (Fig. 1D).

Gpd1R270E is targeted to peroxisomes in *gpd1* cells by its PTS2 (Fig. 1E) but deletion of its PTS2, i.e. ΔN-Gpd1R270E, blocks localisation to peroxisomes in WT cells. This is in contrast to ΔN-Gpd1 which is piggy-back imported with endogenous Gpd1 (Fig. 1E). ΔN-Gpd1 is not imported when endogenous Gpd1 is replaced by Gpd1R270E (Fig. S1D). These data show that Gpd1 dimer formation and piggy-back import are disrupted by the R270E mutation. Furthermore, it illustrates that Gpd1 can be imported both as a monomer and a dimer. Subsequently, we introduced the E195R mutation into Gpd1R270E, in the anticipation that this may restore homodimerisation. Using the above described piggy-back import assay, we tested various combinations of ΔN-Gpd1 mutants with either WT or dimerization mutants in Gpd1. For instance, ΔN-Gpd1E195R/R270E-GFP fails to be co-imported with endogenous Gpd1 (Fig. 1F, panel1) but is imported upon expression of Gpd1E195R, R270E-mCherry (Fig. 1F). In line with the piggy-back import assay results, co-immunoprecipitation experiments revealed that Gpd1E195R/R270E-HA forms a stable dimer with Gpd1E195R/R270E-GFP but not with WT Gpd1-GFP (Fig. S1E). Furthermore, Gpd1E195R/R270E-HA behaves the same as Gpd1-HA during gel filtration chromatography, indicating that the mutations do not lead to aggregation (Fig. S2). An overview of the results from the co-import assays is presented in Fig. 1G. We conclude that Gpd1 can be imported as a dimer, but dimer formation is not a prerequisite for import.

**Figure 2:**
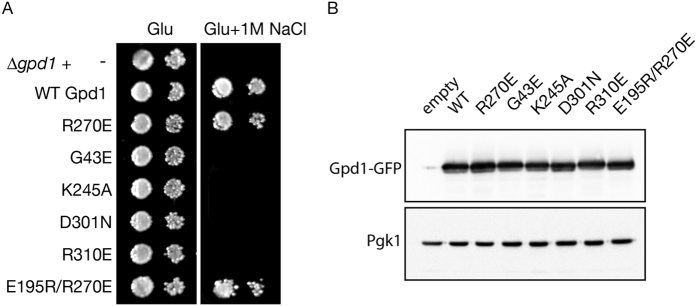
Disruption of Gpd1 homodimerisation does not affect growth under salt stress. (**A**) *gpd1* cells were transformed with Gpd1-GFP expression plasmids encoding WT and various mutants. Cells from log phase cultures were spotted on Glucose medium (Glu) in the presence and absence of 1M NaCl and (**B**) analysed by immunoblotting. Gpd1-GFP was detected by anti-GFP and Pgk1 was used as loading control.

Whereas *gpd1* cells are unable to grow on high salt containing medium, expression of *Gpd1-GFP* under control of the *GPD1* promoter rescues the growth defect (Fig. 2A). Using the ternary complex of human GPD1 with NAD^+^ and dihydroxyacetone (1WPQ)^42^, as a guide, single residue mutations in the *S. cerevisiae* enzyme were designed that were predicted to interfere with NAD^+^ binding. The NAD^+^ pyrophosphate packs against G43 and makes electrostatic interactions with R310. Replacing these residues with glutamate (G43E and R310E) would introduce adverse electrostatic interactions with the cofactor and, in the case of G43E, additional adverse steric effects. Similarly, the active site residues D301 and K245 interact with the substrate and replacing these residues with asparagine or alanine, respectively (D301N and K245A), might be expected to have a deleterious effect on substrate binding. All of these single mutants (G43E, R310E, D301N and K245A) do not complement growth on high salt. In contrast, expression of Gpd1 R270E restores growth on high salt media implying it is a functional enzyme. All mutants are stably expressed (Fig. 2B). We conclude that formation of a stable homodimer is not a prerequisite for Gpd1 function.

### The PTS1 pathway doesn’t support Gpd1-PTS1 dimer import

**Figure 3:**
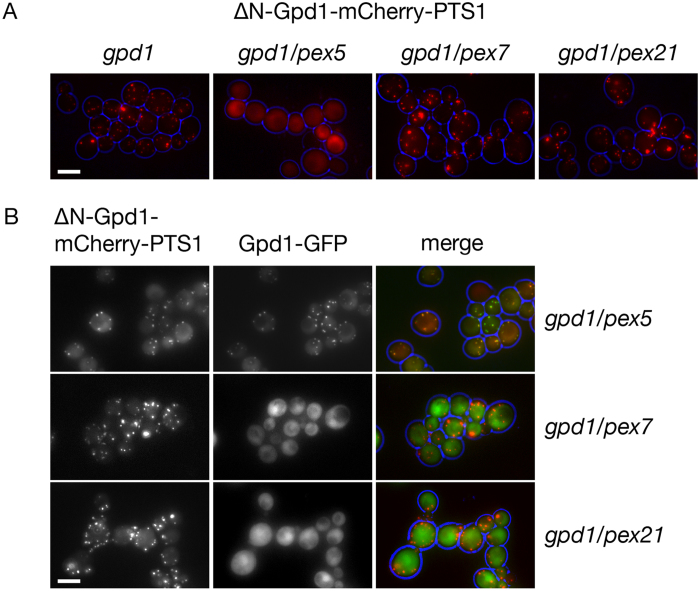
The PTS1 pathway doesn’t support Gpd1-PTS1 dimer import. (**A,B**) Localisation of ΔN-Gpd1-mCherry-PTS1 (**A,B**) and Gpd1-GFP (**B**) in strains indicated. Fluorescence microscopy of cells from cultures in log phase. All versions of Gpd1 were expressed from centromeric plasmids under control of the endogenous promoter. Bar, 5 μm.

To test whether co-import of Gpd1 can be redirected via the PTS1 pathway, we replaced the N-terminal PTS2 with a C-terminal PTS1 (ΔN-Gpd1-mCherry-PTS1). As expected this fusion protein is imported in *gpd1* cells (Fig. 3A) and in mutants blocked in the PTS2 pathway (*pex7/gpd1* and *pex21/gpd1*) but not in *pex5/gpd1* cells where PTS1 import is blocked. Import of ΔN-Gpd1-mCherry-PTS1 in *pex5/gpd1* cells is restored upon co-expression of Gpd1-GFP (Fig. 3B). This indicates that Gpd1-GFP can support co-import of ΔN-Gpd1-mCherry-PTS1 via the PTS2 import pathway in these cells i.e. that neither removal of the PTS2 nor addition of mCherry-PTS1 blocks dimer formation with Gpd1-GFP in *pex5/gpd1* cells. Surprisingly, although ΔPTS2-Gpd1-mCherry-PTS1 is efficiently imported in *pex7/gpd1* and *pex21/gpd1* cells, it does not support piggy-back import of Gpd1-GFP in *pex7/gpd1* and *pex21/gpd1* cells (Fig. 3B). We conclude that the PTS1 pathway imports redirected Gpd1 as monomers.

### Pnc1 piggy-back import requires Gpd1 to follow the PTS2 pathway

**Figure 4:**
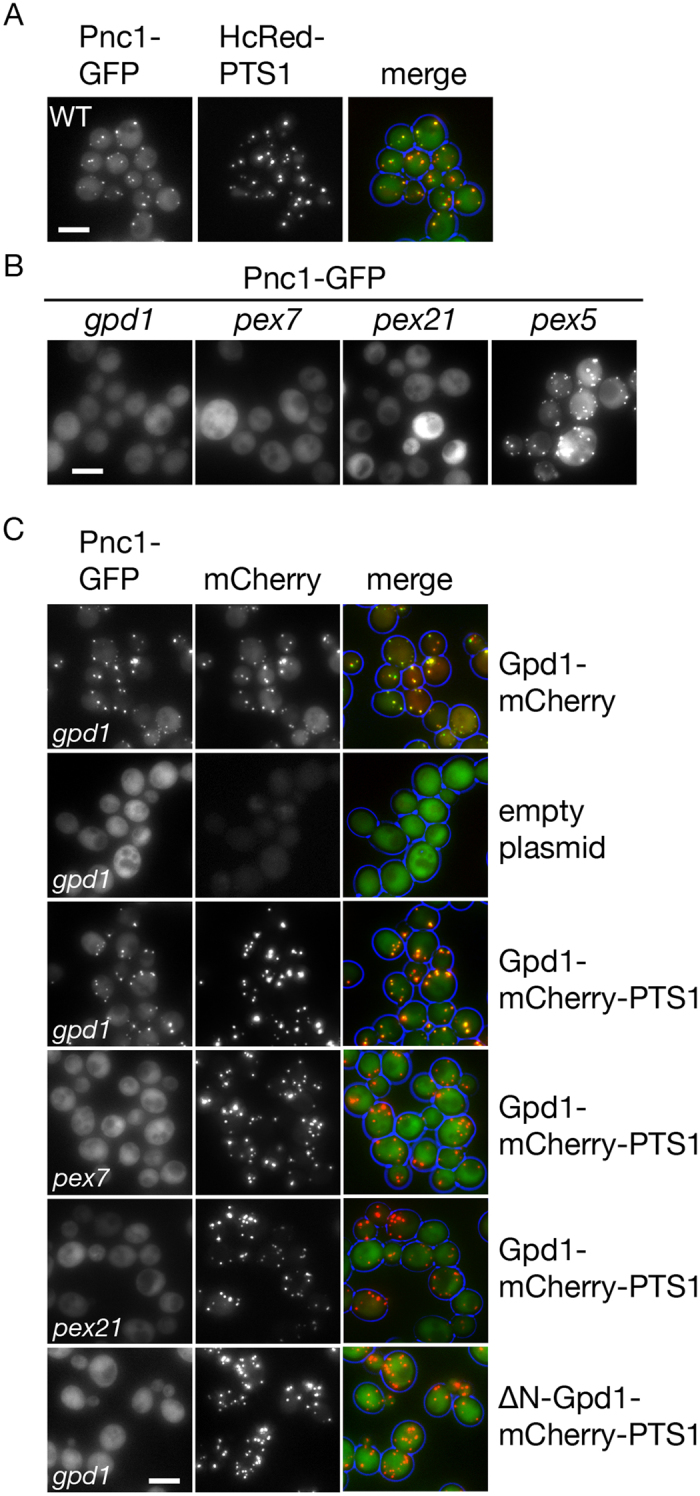
Redirection of Gpd1 to the PTS1 pathway does not support Pnc1 co-import. Localisation of Pnc1-GFP in (**A**) WT cells expressing the peroxisomal marker HcRed-PTS1 and (**B**) in *gpd1, pex7, pex21* and *pex5* cells. (**C**) Localisation of Pnc1-GFP in strains indicated co-expressing Gpd1-mCherry, Gpd1-mCherry-PTS1 and ΔN-Gpd1-mCherry. Fluorescence microscopy of cells from cultures in log phase. All versions of Gpd1 were expressed from centromeric plasmids under control of the endogenous promoter. Pnc1 was tagged in the genome with GFP. Bar, 5 μm.

Pnc1 is partially localised to peroxisomes by piggy-back import with Gpd1^37^,^38^. Indeed, in *gpd1* cells, Pnc1-GFP is mislocalised to the cytosol whereas in a mutant selectively disrupted in the PTS1 pathway, *pex5*, Pnc1 is imported (Fig. 4A,B).

Expression of Gpd1-mCherry restores import of Pnc1-GFP in *gpd1* cells (Fig. 4C). Since Gpd1 is not imported into peroxisomes in *pex7* and *pex21* cells (Fig. 1A), we tested whether Pnc1 can be co-imported when Gpd1 is artificially directed to peroxisomes via the PTS1 pathway. Expression of Gpd1-mCherry-PTS1 in *gpd1* cells supports piggy-back import of Pnc1-GFP into peroxisomes but not in *pex7* and *pex21* cells. Furthermore, deletion of the PTS2 in Gpd1-PTS1 abolished piggy-back import of Pnc1 in *gpd1* cells. Addition of a PTS1 to Gpd1-FP or ΔN-Gpd1-FP results in a more pronounced peroxisomal localization and a decrease in cytosolic labelling^40^ (see also Fig. 4C). This suggests that import of these constructs is more efficient than of Gpd1. However, the failure of Gpd1-PTS1 to support Pnc1 co-import in *pex7* and *pex21* cells is not a consequence of its efficient import in *gpd1* cells, as Gpd1-mCherry-PTS1 is also efficiently imported and Pnc1-GFP is still co-imported (Fig. 4C). Together these observations support a model whereby Pnc1 piggy-back import requires Gpd1 to follow the PTS2 pathway.

### Pnc1 co-import requires Gpd1 to be imported as dimer

**Figure 5:**
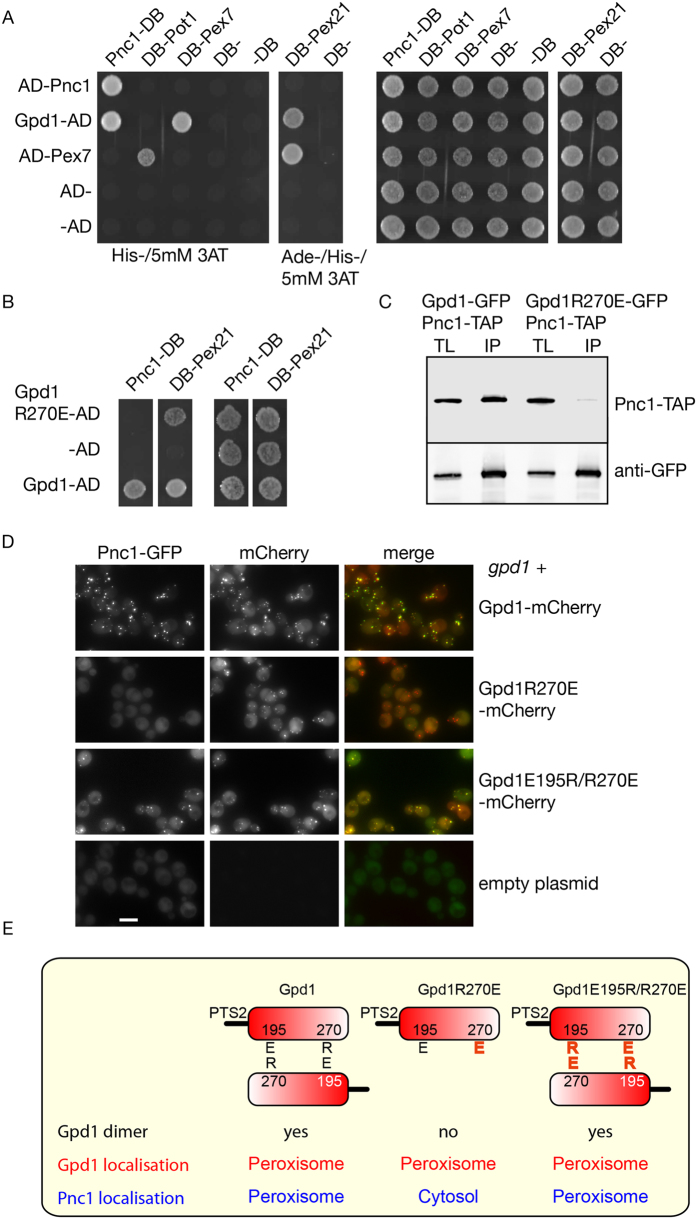
Pnc1 co-import relies on the ability of Gpd1 to form homodimers. (**A,B**) Yeast two hybrid analysis of Pnc1, Gpd1, Gpd1R270E and Pex7 fused to Gal4 transactivation domain with Pnc1, Pot1, Pex7 and Pex21 fused to Gal4 DNA binding domain. Mat A cells transformed with activation domain constructs were mated with Mata cells transformed with DNA binding domain constructs and selected and pinned onto plates. Panel 1 and 2, screening condition was SD His- medium containing 5mM 3AT and SD Ade-, His- medium containing 5 mM 3AT. Panel 3 and 4 are controls for the growths of diploid transformants. (**C**) GFP-TRAP co-immunoprecipitation experiments using cell lysates from *gpd1* cells expressing Pnc1-TAP and either Gpd1-GFP or Gpd1R270E-GFP. Cells were grown on 1M NaCl-containing medium. Proteins were detected by immunoblotting. TL, total lysate; IP, immunoprecipitate. (**D**) Localisation of Pnc1-GFP in *gpd1* cells expressing Gpd1-mCherry, Gpd1R270E-mCherry and Gpd1E195R/R270E-mCherry. (**E**) Schematic representation of the ability of Gpd1, Gpd1R270E and Gpd1E195R/R270E for homodimer formation, and Pnc1 co-import. R and E indicate arginine and glutamic acid residues in Gpd1 that are important for dimer formation. All versions of Gpd1 were expressed from centromeric plasmids under control of the endogenous promoter. Pnc1 was tagged in the genome with GFP. Bar, 5 μm.

The requirements for Pnc1 piggy-back import resemble those of Gpd1 dimer import. This raises the possibility that Pnc1 piggy-back import requires Gpd1 to form a dimer. First, we tested whether Pnc1 and Gpd1 interact using yeast two hybrid as described previously^37^. Pnc1 interacts with itself and with Gpd1 (Fig. 5A). This is in agreement with previous observations^44^. Gpd1 was also found to interact with Pex7 and Pex21. No interaction was observed between Pnc1 and Pex7, Pex21. The Pnc1-Gpd1 interaction is abolished in the Gpd1 dimerisation mutant (Fig. 5B). We performed co-immunoprecipitation experiments to test whether Pnc1 and Gpd1 are part of a stable complex. Indeed, WT Gpd1-GFP is co-precipitated with Pnc1-TAP but not with Gpd1R270E-GFP (Fig. 5C).

The lack of interaction between Pnc1 and the dimerisation mutant of Gpd1-mCherry is relevant to Pnc1 localisation as Pnc1-GFP was not co-imported with the Gpd1 dimerisation mutant (Fig. 5D). These results are compatible with either the model that Pnc1 binds the Gpd1 dimer before or during its targeting to peroxisomes or that the R270E mutation disrupts binding of Pnc1 to monomeric Gpd1. To discriminate between these explanations, we determined the localization of Pnc1-GFP in cells expressing Gpd1E195R/R270E. As shown above, this double mutant restores Gpd1 homodimerisation. The double mutant also restored Pnc1 piggy-back import into peroxisomes (Fig. 5D). We conclude that stable Gpd1 dimers are required for Pnc1 piggy-back import.

**Figure 6:**
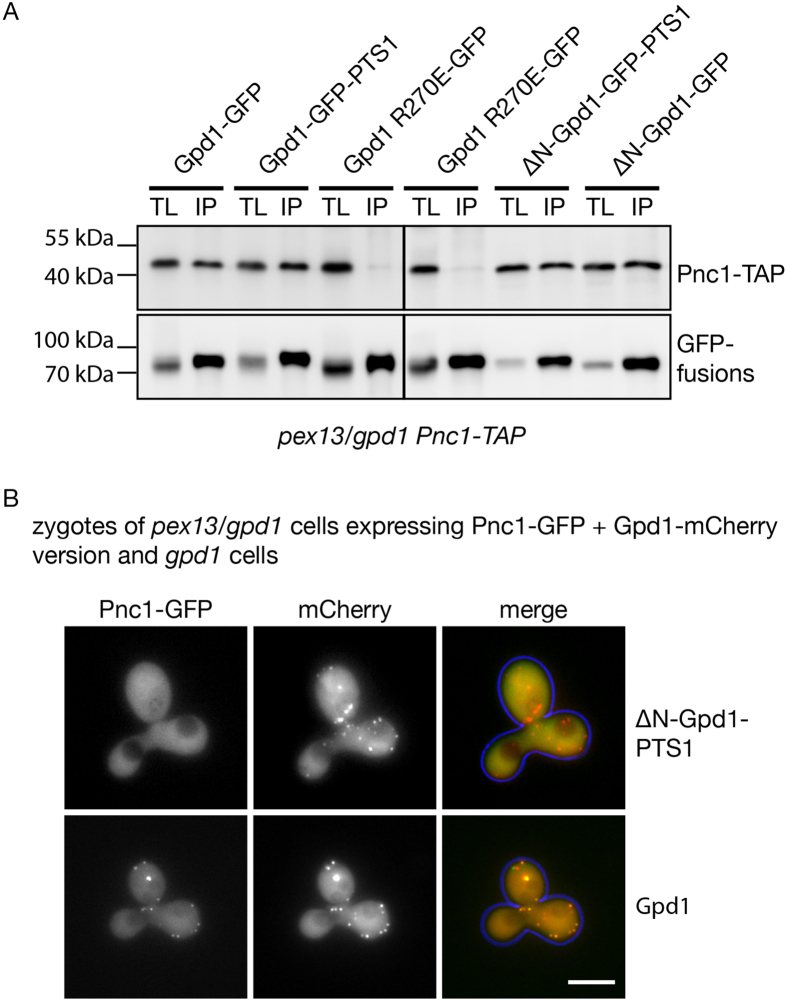
The lack of Pnc1 co-import via the PTS1 pathways is not caused by an inability to bind Gpd1-PTS1 in the cytosol. (**A**) GFP-TRAP co-immunoprecipitation experiments using cell lysates from *pex13/gpd1* cells expressing Pnc1-TAP and either Gpd1-GFP, Gpd1-GFP-PTS1, ΔN-Gpd1-GFP-PTS1, ΔN-Gpd1-GFP or Gpd1R270E-GFP. Cells were grown on 1 M NaCl-containing medium. Proteins were detected by immunoblotting. TL, total lysate; IP, immunoprecipitate. (**B**) Localisation of Pnc1-GFP and mCherry tagged Gpd1 or ΔN-Gpd1-GFP-PTS1 in zygotes formed after mating of *gpd1* cells with *pex13/gpd1* cells expressing Pnc1-GFP tagged in the genome and either Gpd1-mCherry or ΔN-Gpd1-PTS1. Expression of Gpd1 versions was controlled by the *GAL1/10* promoter. Expression was induced for 2 h on galactose medium. Cells were subsequently chased on glucose medium for 2 h to shut down expression, before they were mated with *gpd1* cells on glucose medium. Images were acquired 2 to 3 h after initiation of mating. Bar, 5 μm.

Since neither Gpd1 dimer import nor Pnc1 piggy-back import are supported by the PTS1 pathway, we tested by co-immunoprecipitation experiments, whether addition of a PTS1 to Gpd1 interferes with the formation of the Pnc1-Gpd1 complex in the cytosol. For this we used *pex13/gpd1* cells, where matrix protein import is blocked and both proteins localise to the cytosol. A similar amount of Pnc1-TAP was precipitated with Gpd1-GFP and Gpd1-GFP-PTS1 (Fig. 6A). Subsequent removal of the PTS2 (ΔN-Gpd1-PTS1) does not interfere with the formation of a Gpd1-GFP-PTS1/Pnc1 complex either. Gpd1R270E was used as negative control (Fig. 6A). Since Gpd1-PTS1/Pnc1 complexes form in *pex13/gpd1* cells, we tested whether these complexes could be imported upon complementation. For this we used a mating assay^45^. Pnc1-GFP expressing *pex13/gpd1* cells were pulse-labelled with either mCherry-tagged ΔN-Gpd1-PTS1 or Gpd1, chased for 2 hr, and then mated with *gpd1* cells of the opposite mating type. Two hours later, zygotes were imaged. This allowed us to follow the pool of pulse mCherry-labelled proteins present in the cytosol of *pex13/gpd1* cells and to monitor their fate after import into peroxisomes was restored. Moreover, we can then monitor the consequence of this on Pnc1-GFP localisation. In line with the result presented in Fig. 4, in zygotes expressing Gpd1-mCherry both Pnc1-GFP and Gpd1 localised to peroxisomes (Fig. 6B). In contrast, in zygotes expressing ΔN-Gpd1-PTS1, Pnc1-GFP remained cytosolic. We conclude that although Pnc1 can interact with ΔN-Gpd1-PTS1 in the cytosol, these complexes are not imported via the PTS1 pathway.

## Discussion

Our study supports a model whereby Gpd1 forms dimers in the cytosol to which Pnc1 binds before they are co-imported via the PTS2 import pathway. Gpd1 is an abundant homodimeric protein with partial localisation to peroxisomes, the cytosol and the nucleus. The dimer is stabilised by interactions between E195 and R270. Disruption of the dimer was achieved by substituting the arginine residue 270 for glutamate (R270E) and restored by subsequently converting glutamate 195 for arginine. Using these mutants we found that Gpd1 monomers can be imported via the PTS2 pathway, indicating that dimerisation is not a prerequisite for import. Redirecting the protein to the PTS1 pathway did restore import in *pex7* and *pex21* cells, but it did not allow for dimer import. This is an intriguing result as both the PTS1 and PTS2 pathway have been shown to accommodate oligomeric proteins (see for instance in refs 46, 47, 48, 49).

Some proteins lacking a PTS1 and PTS2 oligomerise with PTS-containing proteins to enter peroxisomes. For instance, in mammals, SOD and LDH subunit A oligomerise and are co-imported with PTS1-containing CCS1 and LDH B, respectively^32^,^33^. No natural PTS-lacking protein has been shown to piggy-back onto a *S. cerevisiae* PTS1 protein. During the course of this study Pnc1 was shown to be co-imported with Gpd1, a PTS2 containing protein^37^,^38^. Our results confirmed this as Gpd1 is required for Pnc1 import via the Pex7, Pex21 import pathway and Pnc1 interacts with Gpd1 in a yeast two hybrid assay and by co-immunoprecipitation. Of particular interest is our observation that the Gpd1R270E mutant which does not form homodimers *in vivo* is unable to (1) form a stable complex with Pnc1 as assayed by yeast two hybrid and co-ip experiments and (2) support piggy-back import of Pnc1. Pnc1 co-import is restored by introduction of a second mutation that now reforms the ion paired interaction in the inverse orientation between two Gpd1 monomers. Upon redirection of Gpd1 from the PTS2 pathway to the PTS1 pathway, Pnc1 is unable to follow, another condition where Gpd1 dimer import is affected. We conclude that Gpd1 needs to be imported as a dimer via the PTS2 pathway to support Pnc1 piggy-back import.

Although peroxisomal enzymes can fold and assemble into oligomers in the cytosol before they are imported, *in vitro* import studies suggest that, at least for several PTS1 proteins, monomer import occurs more efficiently^30^,^31^. In addition, several oligomeric proteins assemble inside peroxisomes^50^,^51^.

The kinetics of oligomerisation appears to affect import *in vivo*. For instance, soluble epoxide hydrolase is a dual localised protein that resides both in the cytosol and peroxisomal matrix. A polymorphism strongly enhances its peroxisomal localisation by destabilising SEH dimers, which have been proposed to affect PTS1-receptor recognition^52^,^53^. The opposite effect has been described for alanine:glyoxylate aminotransferase (AGT). Here a polymorphism strengthens a weak mitochondrial targeting signal. This combined with a mutation that decreases dimer stability results in mislocalisation from peroxisomes to mitochondria^54^,^55^,^56^.

Gpd1 on the other hand is mainly localised to the cytosol and nucleus with only a minor amount to peroxisomes. Disruption of dimer formation does not appear to affect this distribution, suggesting that the control of localisation is at a different level, maybe recognition of the PTS2 or the capacity of the PTS2 import pathway. Swapping the PTS2 for a PTS1 does results in a change in its distribution, with most Gpd1 now located to peroxisomes (ref. 40; see also Figs 3A and 4C).

Interestingly, although the PTS1 pathway can mediate the import of Gpd1-PTS1, it is unable to support homodimer import and Pnc1 piggy-back import (Figs 3B, 4C and 6B). This is not because Gpd1 dimers and Gpd1/Pnc1 complexes can’t form (Figs 3B and 6A). Several scenarios come to mind to explain why Gpd1-PTS1 dimers and Gpd1-PTS1/Pnc1 complexes are not imported; (1) Pex5 only recognises the PTS1 when Gpd1-PTS1 is a monomer, (2) Pex5 binding to Gpd1-PTS1 results in disassembly of the complexes, (3) Pex5 does recognise the complexes but can’t deliver the cargo to the peroxisomal matrix.

With respect to the first scenario, we tagged Gpd1 with either GFP or mCherry attached to a seven glycine-alanine linker and fused an extended PTS1 (PLHSKL) to the C-terminus of these tags. We anticipate that this would offer enough flexibility for Pex5 to recognise the PTS1. Although these tags do not interfere with function and dimer formation of Gpd1, when one considers that the N- and C-termini of GFP or mCherry are in proximity of each other and that the C-terminus of Gpd1 is not far away from the dimer interphase, it remains possible that the PTS1 is not exposed in the Gpd1-FP-PTS1 dimer. Hence, only monomers are delivered to peroxisomes. Alternatively, Pex5 does recognise the PTS1 in Gpd1-PTS1 complexes but binding results in a conformational change that leads to dissociation of Gpd1-PTS1 and Pnc1 (scenario 2). With respect to scenario 3, since in *S. cerevisiae* PTS1 and PTS2 proteins are imported via distinct import channels^16^,^17^,^24^, it is tempting to speculate that these channels may display an unanticipated selectivity towards certain oligomeric proteins.

When considering the co-import process of Pnc1, it is important to note that Pnc1 can assemble into homomultimers *in vitro* and *in vivo*^35^,^36^. Pnc1p self-association is thought to occur through the sequential addition of monomers. Whether Gpd1 co-imports Pnc1 monomers or multimers and whether Pnc1 functions as a homomultimer in the peroxisomal matrix is unclear. It would be of interest to further investigate the oligomeric status of Pnc1 both during its piggy-back import into peroxisomes and inside peroxisomes. Future investigations will focus on dissecting the underlying mechanism of this hetero-oligomeric import process.

## Materials and Methods

### Yeast strains, media and growth conditions

Cells were grown at 30 °C in either of the following media: YPD (1% yeast extract, 2% peptone, 2% glucose), minimal media (YM2) for the selection of the uracil (URA3) prototrophic marker (2% glucose, 0.17% yeast nitrogen base without amino acids, 0.5% ammonium sulphate, 1% casamino acids) or minimal media (YM1) for the selection all prototrophic markers except uracil (2% glucose, 0.17% yeast nitrogen base without amino acids, 0.5% ammonium sulphate).

*S. cerevisiae* strains used in this study are listed in supplementary information Table S1. BY4741 or BY4742 wild type strains and deletion mutant marked with KanMX cassette were obtained from Euroscarf. Any strains created in this study were modified from the BY4741 or BY4742 using the HphMX4 cassette^57^ and selected on YPD containing 300 μg/ml Hygromycin B (Melford, UK). Before imaging, cells were grown overnight on selective glucose medium and the following morning cells were diluted to OD_600_ = 0.1 in selective glucose medium and incubated on an orbital shaker until logarithmic phase before imaging.

### Mating assay

For mating, Gpd1 constructs were induced by growth on selective galactose medium for overnight. Expression was shut down by switching cells to selective YPD medium for 2 h. 0.25 × 10^7^ cells of each mating partner were mixed in a microfuge tube and collected by centrifugation. The cell pellet was resuspended in 50 μl YPD and spotted onto the surface of a YPD plate. After 2 h, cells were scraped of with 1 μl inoculation loop and imaged.

### Plasmids

Most of the plasmids used in this study, were constructed by gap repair^58^ in Ycplac derivatives^59^. The ORF of interest was amplified by PCR containing 18 nucleotides as flanking region identical to each side of the intended insertion site. All *GPD1* expression constructs contained the 523bp upstream from the ATG as its promoter region. GPD1-mCherry-PTS1 was constructed by fusion of mCherry-SKL to the C-terminus of Gpd1. ∆N-Gpd1 was constructed by deletion of the first twenty-one amino acids from the N-terminus and reintroduction of a start codon. Point mutations were generated by site directed mutagenesis. An overview of all plasmids used in this study is presented in supplementary information Tabel S2. Oligonucleotides used for generating plasmids and recombinant strains are presented in supplementary information Tabel S3. Pnc1 was tagged in the genome with GFPS65T^60^ or TAP tag (Open Biosystems).

### Image acquisition

Image acquisition and processing was performed as described previously^61^. Cells were analysed with a microscope (Axiovert 200 M; Carl Zeiss MicroImaging, Inc.) equipped with an Exfo X-cite 120 excitation light source, band pass filters (Carl Zeiss MicroImaging, Inc. and Chroma Technology Corp.), an α Plan-Fluar 100x/1.45 NA, Plan-Apochromat 63x/1.4 NA or A-plan (Carl Zeiss MicroImaging, Inc.), and a digital camera (Orca ER; Hamamatsu). Image acquisition was performed using Volocity software (Improvision). Fluorescence images were collected as 0.5 μm Z-stacks using exposures of up to 200 ms, merged into one plane in Openlab, and processed further in Photoshop (Adobe). Brightfield images were collected in one plane, and processed where necessary to highlight circumference of the cells.

### Yeast two-hybrid growth assay

A two-hybrid assay was performed by expressing the bait protein as a Gal4p DNA-binding-domain-fusion in PJ69-4α and mated with an array of Gal4p activation-domain-protein fusions, expressed in the PJ69-4a strain^62^,^63^. Plasmids for C-terminal fusion were derivatives of pBDC and pADC^64^. The cells were grown in selective media in 96 well plate overnight. For mating, 25 μl from each mating type was mixed in 150 μl YPD and plates were incubated at 30 °C for two days. After mating, cells were spotted by pinning onto SD minimal media lacking tryptophan and leucine for diploids selection and plates were incubated at 30 °C for two days and transferred to a 96 well plate and grown selectively overnight. Diploids were pinned onto an SD plate lacking tryptophan and leucine, and a SD plate lacking tryptophan, leucine, histidine and for Pex21 interactions, also lacking adenine, supplemented with an appropriate concentration of 3-amino-1, 2, 4-triazole (determined experimentally). The plates were imaged after incubation at 30 °C.

### Co-immunoprecipitation

Cells were grown on selective glucose medium for overnight. The following morning, cells were grown logarithmically before transferring to 0.8 M NaCl selective medium and grown overnight (16 h). Cells were harvested at 2,500 rpm for 5 min and resuspended in pre-cooled 1000 μl 1xHEPES lysis buffer (20 mM HEPES, 100 mM KAc and 5 mM MgAc pH 7.5 with protease inhibitor cocktail (Roche) and 1 mM PMSF. 400 μ l of 425–600 μm glass beads were added and the tubes were pulsed in a mini bead beater (Biospec Products) at full speed for 30 sec for 4 times with 30 sec on ice water between the pulses. The samples were centrifuged at 2500 rpm 4 °C for 1 min. The supernatant was centrifuged at 13,000 rpm for 10 min at 4 °C. 900 μl was transformed into a clean tube and 100 μl was kept as total lysate (TL). GFP-TRAP beads (Chromotek) were washed three times with lysis buffer. Equal amount of beads was added to each sample and left to rotate at 4 °C for an hour. The beads were washed four times with lysis buffer before elution with 75 μl 1xHEPES buffer, 25 μl of 4x loading dye and heating at 95 °C for 5 min. the samples were centrifuged at 13,000 rpm for 1 min in before loading to SDS-PAGE gel.

### Immunoblotting

For preparation of extracts by alkaline lysis, cells were centrifuged and pellets resuspended in 0.2 M NaOH and 0.2% β-mercaptoethanol and left on ice for 10 min. Soluble protein was precipitated by addition of 5% TCA for a further 10 min. Following centrifugation (13 000 *g*, 5 min, 4 °C), soluble protein was resuspended in 10 μl 1 M Tris–HCl (pH 9.4) and boiled in 90 μl 1 × SDS–PAGE sample loading buffer for 10 min. Samples (0.25–1 OD_600_ equivalents) were resolved by SDS–PAGE followed by immunoblotting. Monoclonal anti-GFP antibody was obtained from Roche (11814460001), peroxidase-anti-peroxidase was obtained from Sigma (P 1291), and anti-Pgk1 22C5 from Invitrogen. Secondary antibody was HRP-linked Goat anti-mouse polyclonal (BioRad 1706516). Blots were blocked in 2% (w/v) fat-free Marvel™ milk in TBS-Tween 20 (50 mM Tris-HCl (pH 7.5), 150 mM NaCl, 0.1% (v/v) Tween 20). Immunoreactive proteins were detected by enhanced chemiluminescence (Biological Industries) and blots were imaged with a G:box (Syngene). Blot images were imported as uncompressed TIFF files into Adobe Photoshop and where necessary, levels were adjusted across the whole blot. Full blots are shown in Supplementary information, strips of the blots are shown in figures.

## Additional Information

**How to cite this article**: Saryi, N. A. A. *et al*. Pnc1 piggy-back import into peroxisomes relies on Gpd1 homodimerisation. *Sci. Rep.* **7**, 42579; doi: 10.1038/srep42579 (2017).

**Publisher's note:** Springer Nature remains neutral with regard to jurisdictional claims in published maps and institutional affiliations.

## Supplementary Material

Supplementary Information
