# Supplementary material for: Pnc1 piggy-back import into peroxisomes relies on Gpd1 homodimerisation
Source: Sci Rep. 2017 Feb 17;7:42579. doi: 10.1038/srep42579 (PMC5314374; doi:10.1038/srep42579)

Supplementary information

Pnc1 piggy-back import into peroxisomes relies on Gpd1 homodimerisation

Nadal A. Al Saryi, John D. Hutchinson, Murtakab Y Alhejjaj, Svetlana Sedelnikova, Patrick Baker and Ewald H. Hettema*

*corresponding author, e.hettema@sheffield.ac.uk

Department of Molecular Biology and Biotechnology
University of Sheffield
Firth Court, Western Bank
Sheffield S10 2TN
United Kingdom

Figure S1

Import of Gpd1 dimers.

A) Gpd1-GFP localisation in *S. cerevisiae* WT, *pex5*, *pex7* and *pex21* cells expressing the peroxisomal marker HcRed-PTS1.

(B,C) The dimer of human glycerol-3-phosphate dehydrogenase (1WPQ). A close up cartoon representation in the same orientation as Figure 1, highlighting the ion-pair interaction between R229 from one subunit (pink) and E163 and E254 from the other (green), side chains drawn as sticks (oxygen, red; nitrogen, blue). (D) Localisation of ΔN-Gpd1-GFP in *S. cerevisiae gpd1* cells also expressing Gpd1 and Gpd1R270E from a centromeric plasmid under control of endogenous promoter (pGPD1 and pGPD1R270E, respectively). (E) Schematic representation of the localisation of Gpd1-mCherry and ΔN-Gpd1-GFP mutants in *gpd1* cells co-expressing the various combinations. Red, PTS2-containing Gpd1 versions. Blue, PTS2-lacking versions. R and E indicate arginine and glutamic acid residues in Gpd1 that are important for dimer formation. Fluorescence microscopy of cells from cultures in log phase. Bar, 5 µm.

Figure S2

Gpd1E195R/R270E does not form aggregates. Cell free extracts (CFE) of *gpd1* cells expressing either Gpd1-HA or Gpd1E195R/R270E-HA were analysed by gel filtration using a Superdex 200GL column (10x300 mm) on an AKTA purifier system at constant flow rate of 0,5 ml/min in 50 mM Hepes pH 7.5, 150 mM NaCl buffer. 2.5-3 mg protein was loaded in a 0.5 ml fraction. Fractions of 0.5 ml were collected including the void volume. Versions of Gpd1-HA were detected using anti-HA immunoblotting. Chromatographic profiles are comparable between WT and mutant, with no high molecular weight aggregates being apparent. Arrows indicate fractions where control proteins elute in a separate run, with apparent MW indicated. Note that Gpd1-HA is eluting at a lower than expected apparent MW, suggesting weak interactions with column material.

Table 1 *S. cerevisiae* strains used in this study

strain	source
BY4741 MATa *his3-1 leu2-0 met15-0 ura3-0*	Euroscarf
BY4742 MATα *his3-1 leu2-0 lys2-0 ura3-0*	Euroscarf
BY4741 *gpd1::KanMX*	Euroscarf
BY4742 *gpd1:: HphMX4*	This study
BY4741 *pex5::KanMX gpd1:: HphMX4*	This study
BY4741 *pex7::KanMX gpd1:: HphMX4*	This study
BY4741 *pex21::KanMX gpd1:: HphMX4*	This study
BY4741 *gpd1::KanMX PNC1-TAP::His3MX*	Open Biosystems
BY4741 *PNC1-GFP::His3MX6*	This study
BY4741 *gpd1::KanMX PNC1-GFP::His3MX6*	This study
BY4741 *pex7::KanMX PNC1-GFP::His3MX6*	This study
BY4741 *pex5::KanMX PNC1-GFP::His3MX6*	This study
BY4741 *pex21::KanMX PNC1-GFP::His3MX6*	This study
BY4741 *gpd1::KanMX PNC1-TAP::His3MX6 pex13::URA3*	This study
BY4741 *gpd1::KanMX PNC1-GFP::His3MX6 pex13::URA3*	This study
PJ9-4*a trp1-901, leu2-3,112, ura3-52, his3-200, gal4Δ, gal80Δ*, *LYS2::GAL1-HIS3, GAL2-ADE2, met2::GAL7-lacZ*	[62]
PJ9-4□ *trp1-901, leu2-3,112, ura3-52, his3-200, gal4Δ, gal80Δ*, *LYS2::GAL1-HIS3, GAL2-ADE2, met2::GAL7-lacZ*	[62]

Table 2 Plasmids used in this study

Plasmid	Promoter	Description	vector	Source
Ycplac33	---	Empty plasmid *URA3*/centromeric		[59]
Ycplac111	---	Empty plasmid *LEU2*/centromeric		[59]
pAS63	*HIS3*	*Hc-RED-PTS1*	Ycplac111	[65]
pGAD424	*ADH1*	*GAL4* activation dom. for n-term. fusion		clontech
pGBT9	*ADH1*	*GAL4* DNA bind. dom. for n-term. fusion		clontech
pBDC	*ADH1*	*GDBD* for c-term. fusion		[64]
pADC	*ADH1*	*GAD* for c-term. fusion		[64]
PNA032	*GPD1*	*GPD1-mCherry*	Ycplac33	This study
PNA033	*GPD1*	*GPD1-GFP*	Ycplac33	This study
PNA052	*ADH1*	*GAD-PNC1*	pGAD424	This study
PNA055	*ADH1*	*GDBD-PEX7*	pGBT9	This study
PNA058	*ADH1*	*GDBD-POT1*	pGBT9	This study
PNA035	*GAL1/10*	*mCherry-ΔN-GPD1-PTS1*	Ycplac111	This study
PNA063	*GAL1/10*	*Gpd1-mCherry*	Ycplac111	This study
PNA079	*ADH1*	*GPD1-GAD*	pADC	This study
PNA082	*ADH1*	*GDBD-PEX21*	pGBT9	This study
PNA083	*ADH1*	*PNC1-GDBD*	pDBC	This study
PNA089	*GPD1*	*GPD1* R270E-*mCherry*	Ycplac33	This study
PNA096	*GPD1*	*GPD1 R270E-GFP*	Ycplac111	This study
PNA098	*ADH1*	*GPD1 R270E-GAD*	pADC	This study
PNA101	*GPD1*	*GPD1* R270E-*GFP*	Ycplac33	This study
PNA102	*GPD1*	*GPD1* K254A-*GFP*	Ycplac33	This study
PNA103	*GPD1*	*GPD1* D301N-*GFP*	Ycplac33	This study
PNA104	*GPD1*	*GPD1* G43E-*GFP*	Ycplac33	This study
PNA105	*GPD1*	*ΔN-GPD1-mCherry*	Ycplac33	This study
PNA106	*GPD1*	*ΔN-GPD1 R270E-mCherry*	Ycplac33	This study
PNA107	*GPD1*	*GPD1* R270E, E195R-*mCherry*	Ycplac33	This study
PNA112	*GPD1*	*ΔN-GPD1* R270E, E195R-*GFP*	Ycplac111	This study
PNA115	*GPD1*	*GPD1* R310E-*GFP*	Ycplac33	This study
PNA116	*GPD1*	*GPD1* R270E, E195R-*GFP*	Ycplac33	This study
PNA121	*GPD1*	*GPD1* R270E-HA	Ycplac111	This study
PNA122	*GPD1*	*GPD1* R270E, E195R-HA	Ycplac111	This study
PNA123	*GPD1*	*GPD1*-HA	Ycplac111	This study
PNA125	*GPD1*	*GPD1-mCherry-SKL*	Ycplac33	This study
PNA126	*GPD1*	*GPD1-GFP*	Ycplac111	This study
PNA127	*GPD1*	*ΔN-GPD1-GFP*	Ycplac111	This study
PNA128	*GPD1*	*ΔN-GPD1-mCherry-SKL*	Ycplac33	This study

Table 3 Oligonucleotides used in this study

	DNA sequence 5'-3'	Description
2388	ATAAGGTTAAGGAAGAGTTGAAGGCCCACAACATCAATGTCGTGGATAA ACGGATCCCCGGGTTAATTAA	Forw, C- term. tagging of *PNC1* in the genome
2389	CATTTGCAAGCCACCCTAGTTCATCAGGTTGAAGAAGTATTATTCAGCTC GCATAGGCCACTAGTGGATC	Rev, C- term. tagging of *PNC1* in the genome
2460	ACGTTGTAAAACGACGGCCAGTGAATTCCAAGACAGGGTCAATGAGAC	Forw, GPD1 promoter
2675	GGATCCTCTAGAGTCGACCTGCAGATGGTGAGCAAGGGCGAG	Forw, *mCherry-PTS1* behind *GPD1*.
2676	GATAACACAGGCGGGATCAAGCTTCTACAGTTTACTGTGCAGTGGCTTG TACAGCTCGTCCATG	Rev, *mCherry-PTS1* behind *GPD1*.
2730	GACAGTTGACTGTATCGCCGGAATTCATGCTCAGATATCATATGCAAG	Forw, *PEX7* in pGBT9
2731	GGCTGCAGGTCGACGGATCCTCAACCTAAGCCGTTCCATA	Rev. *PEX7* in pGBT9
2736	GACAGTTGACTGTATCGCCGGAATTCATGTCTCAAAGACTACAAAGT	Forw. *POT1* in pGBT9
2737	GGCTGCAGGTCGACGGATCCCTATTCTTTAATAAAGATGGCG	Rev. *POT1* in pGBT9
2909	CAAACCCAAAAAAAGAGATCGAATTCATGCTCAGATATCATATGCAAG	Forw. *PEX7* in pGAD424
2910	CTCTGCAGGTCGACGGATCCTCAACCTAAGCCGTTCCATA	Rev. *PEX7* in pGAD424
2915	GACAGTTGACTGTATCGCCGGAATTCATGCCCAGTGTCTGCCATAC	Forw. *PEX21* in pGBT9
2916	GGCTGCAGGTCGACGGATCCTCAATCAAGTATGTCTTTGTG	Rev. *PEX21* in pGBT9
2919	AGCTTGAAGCAAGCCTCGATGAAGACTTTAATTGTTGTTG	Forw. *PNC1* in pBDC.
2920	CAGTAGCTTCATCTTTCGTTATTTATCCACGACATTGATG	Rev. *PNC1* in pBDC.
2923	CCAAGCATACAATCCAAGATGTCTGCTGCTGCTGATAG	Forw. *GPD1* in pADC.
2924	TATCCATCTTTGCAAAGGCCTAATCTTCATGTAGATCTAATTC	Rev. *GPD1* in pADC.
2973	TATATTGTACACCCCCCCCCTCCACAAACACAAATATTGATAATATAAAG CAGCTGAAGCTTCGTACGC	Forw, *GPD1* deletion
2974	CCTCGAAAAAAGTGGGGGAAAGTATGATATGTTATCTTTCTCCAATAAAT GCATAGGCCACTAGTGGATCTG	Rev, *GPD1* deletion
3003	AACGCTTCTGCTGCCATCCAAGAAGTCGGTTTGGGTGAGATCATC	Forw, SDM Gpd1R270E
3004	GATGATCTCACCCAAACCGACTTCTTGGATGGCAGCAGAAGCGTT	Rev, SDM Gpd1R270E
3005	ATCTCCATCTGTGGTGCTTTGGCCAACGTTGTTGCCTTAGGTTGTG	Forw, SDM Gpd1K245A
3006	CACAACCTAAGGCAACAACGTTGGCCAAAGCACCACAGATGGAGAT	Rev, SDM Gpd1K245A
3007	CAAGAGTCTGCTGGTGTTGCTAACTTGATCACCACCTGCGCTGG	Forw, SDM Gpd1D301N
3008	CCAGCGCAGGTGGTGATCAAGTTAGCAACACCAGCAGACTCTT	Rev, SDM Gpd1D301N
3044	TTCAAGGTTACTGTGATTGGATCTGAAAACTGGGGTACTACTATTGCCA	Forw, SDM Gpd1G43E
3045	TGGCAATAGTAGTACCCCAGTTTTCAGATCCAATCACAGTAACCTTGAA	Rev, SDM Gpd1G43E
3086	ACCACCTGCGCTGGTGGTGAAAACGTCAAGGTTGCTAGGCTA	Forw, SDM Gpd1R310E
3087	TAGCCTAGCAACCTTGACGTTTTCACCACCAGCGCAGGTGGT	Rev, SDM Gpd1R310E
3094	AGTCGCTCAAGAACACTGGTCTAGAACAACAGTTGCTTACCACATTC	Forw, SDM Gpd1E195R
3095	GAATGTGGTAAGCAACTGTTGTTCTAGACCAGTGTTCTTGAGCGAC	Rev, SDM Gpd1E195R

Supplementary Figure 1

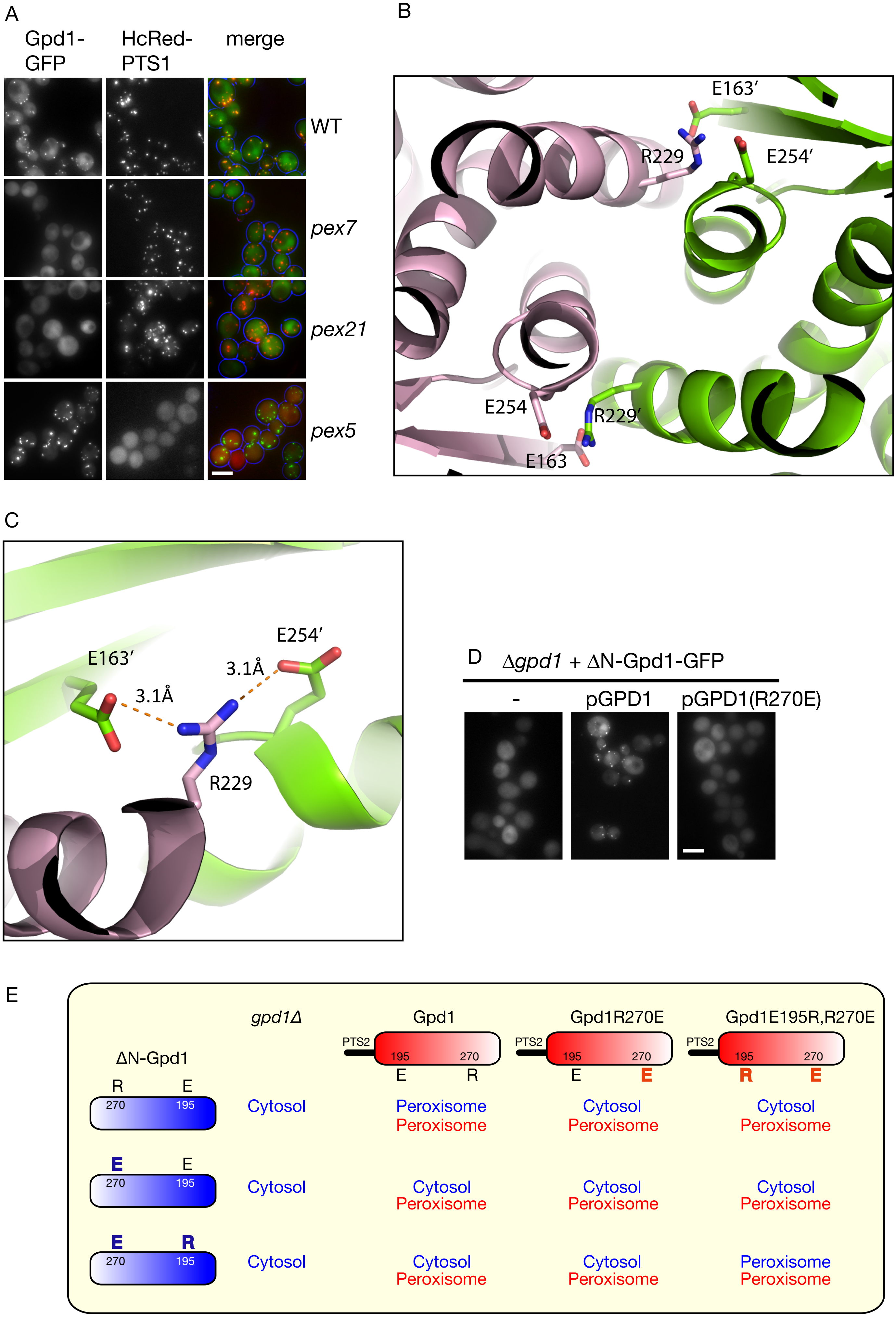

Supplementary Figure 2

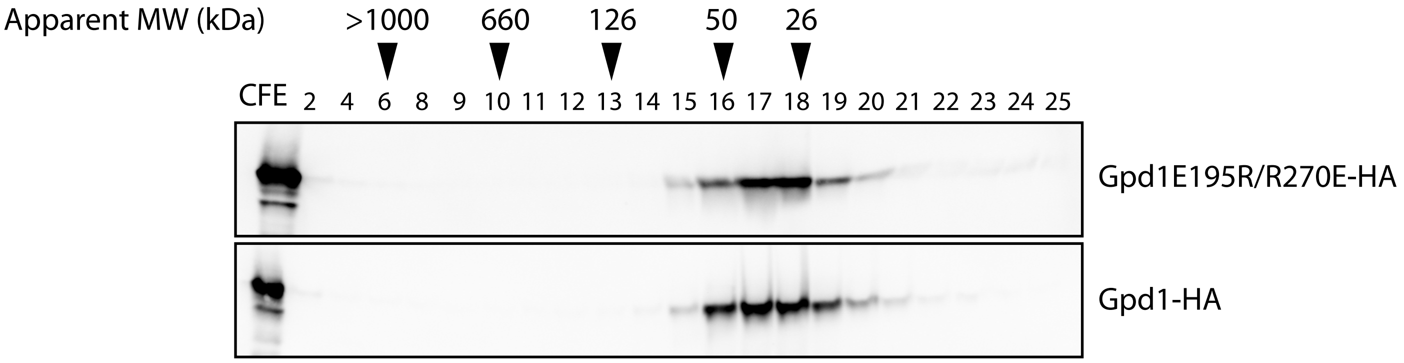

Full blots accompanying figures stated

Fig. 1D

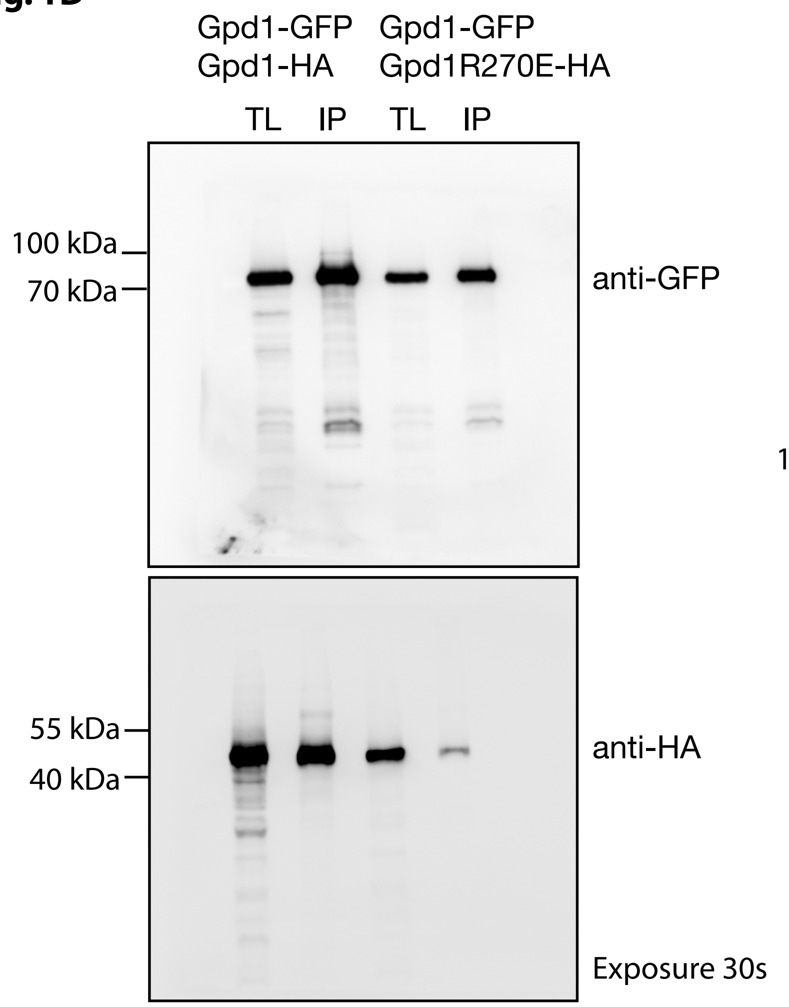

Fig. 1G

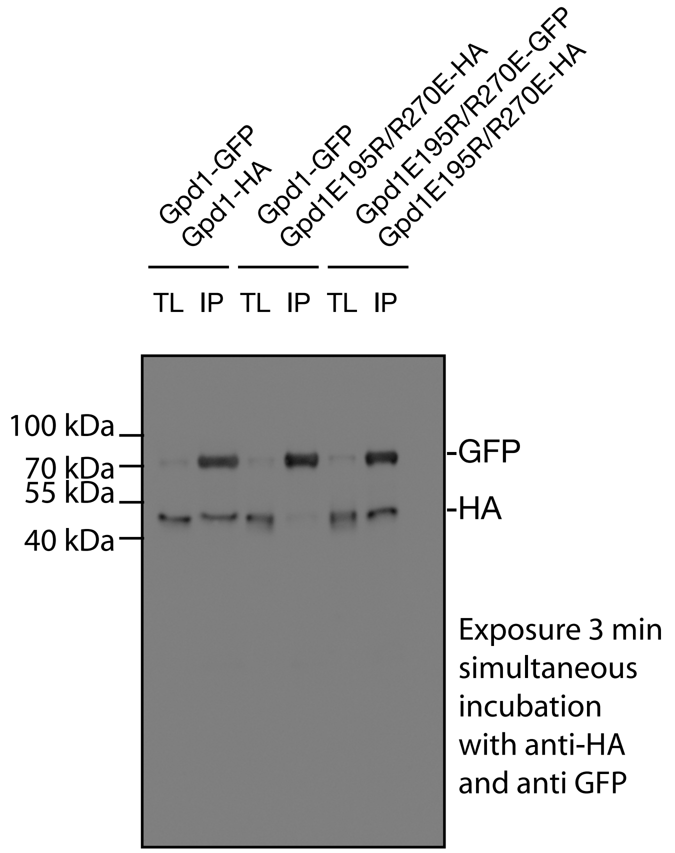

Fig. 2B

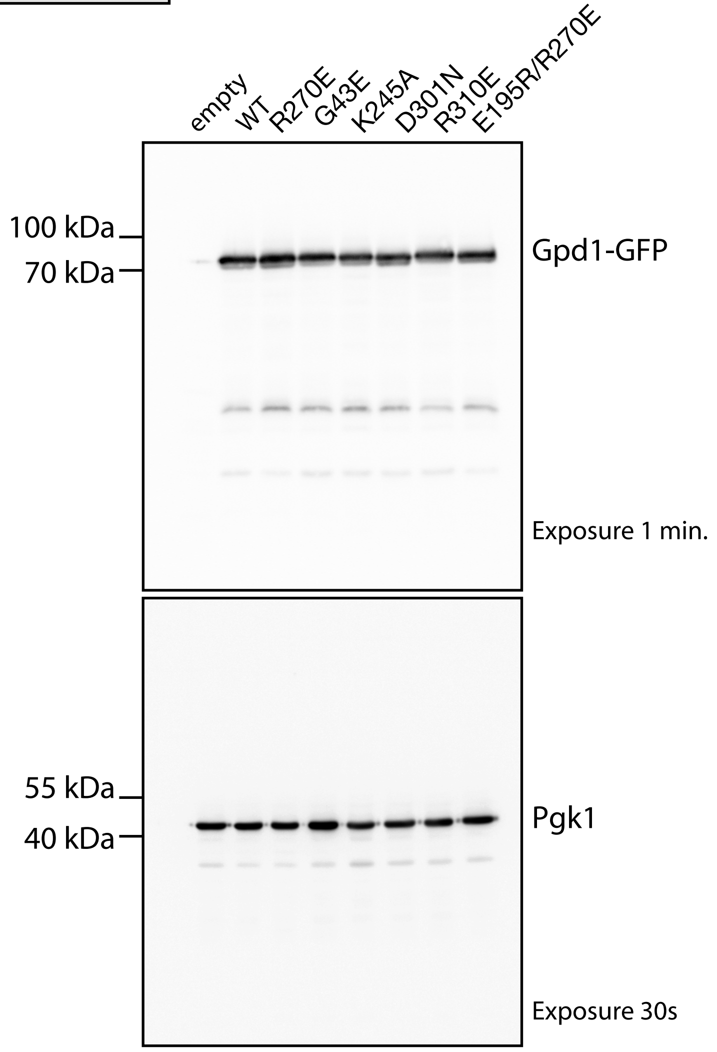

Full blots accompanying figures stated

Fig. 5c

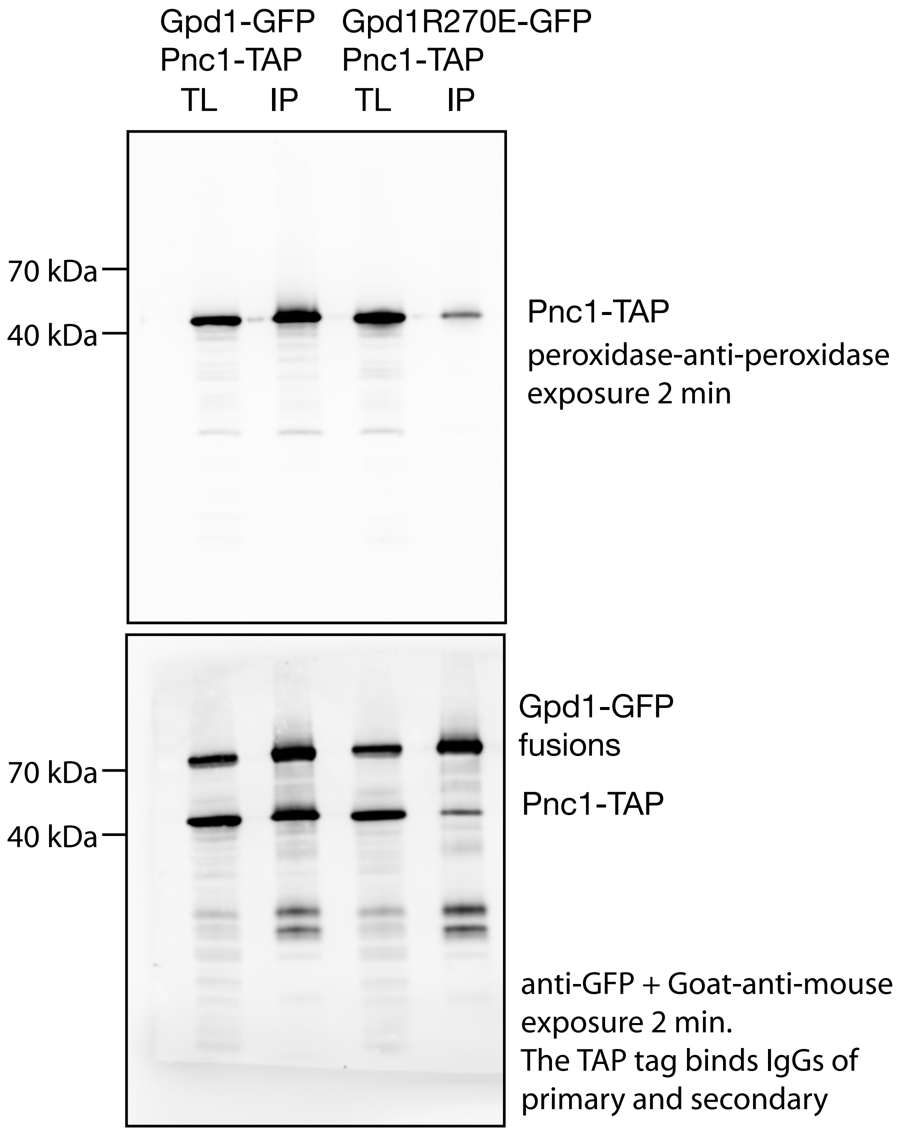

Fig. 6A

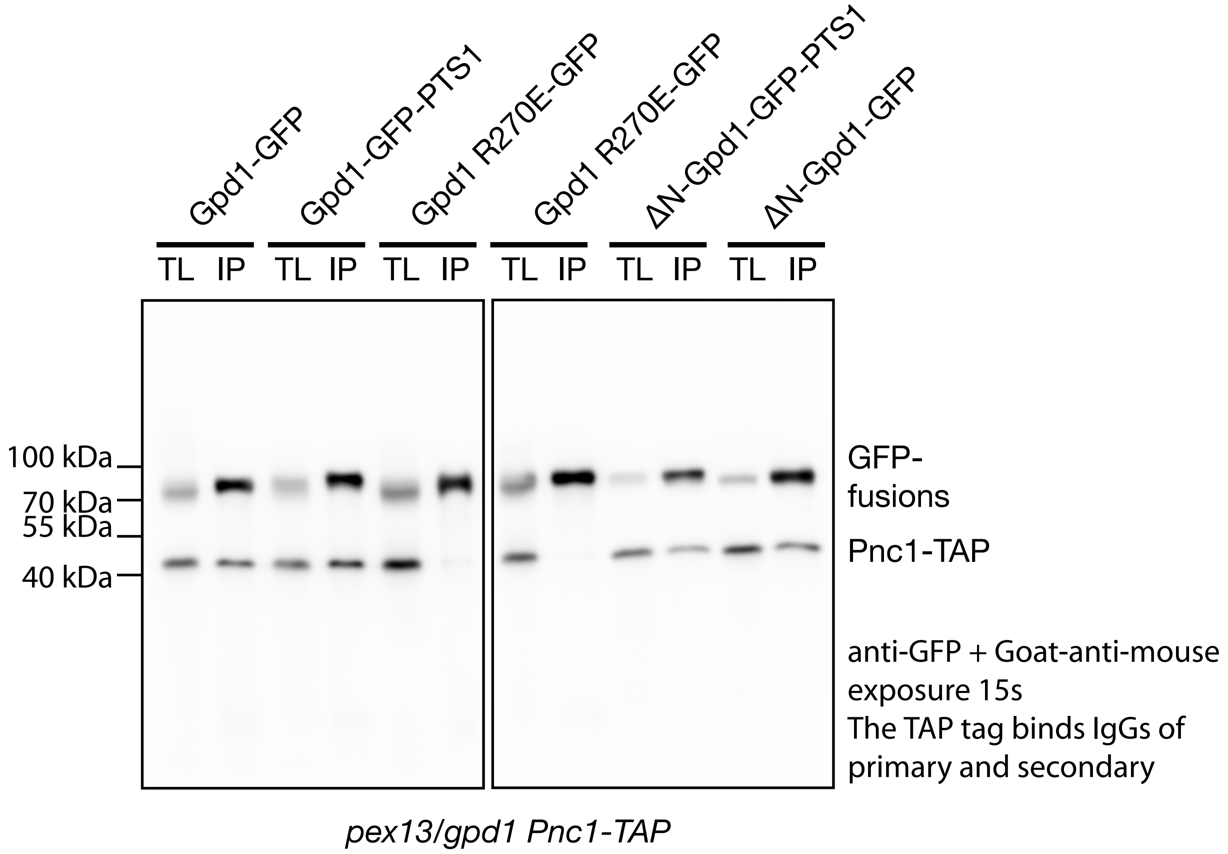

Supplement: Supplementary Information [file srep42579-s1.pdf]
